# Occurrence and Outcome of Infective Endocarditis after Surgical Compared to Transcatheter Pulmonary Valve Implantation in Congenital Heart Disease

**DOI:** 10.3390/jcm13092683

**Published:** 2024-05-02

**Authors:** Alicia Jeanette Fischer, Dominic Enders, Helmut Baumgartner, Gerhard-Paul Diller, Gerrit Kaleschke

**Affiliations:** 1 Department of Cardiology III—Adult Congenital and Valvular Heart Disease, University Hospital Muenster, 48149 Muenster, Germanygerrit.kaleschke@ukmuenster.de (G.K.); 2 Institute of Biostatistics and Clinical Research, University of Muenster, 48149 Muenster, Germany

**Keywords:** congenital heart defect, pulmonary valve, endocarditis, cardiac surgical procedures, heart valve prosthesis

## Abstract

**Background**: Conflicting data exist on the occurrence and outcome of infective endocarditis (IE) after pulmonary valve implantation. Objectives: This study sought to assess the differences between transcatheter pulmonary valve implantation (TPVI) and surgical pulmonary valve replacement (SPVR). **Methods**: All patients ≥ 4 years who underwent isolated pulmonary valve replacement between 2005 and 2018 were analyzed based on the data of a major German health insurer (≈9.2 million insured subjects representative of the German population). The primary endpoint was a composite of IE occurrence and all-cause death. **Results**: Of 461 interventions (cases) in 413 patients (58.4% male, median age 18.9 years [IQR 12.3–33.4]), 34.4% underwent TPVI and 65.5% SPVR. IE was diagnosed in 8.0% of cases during a median follow-up of 3.5 years. Risk for IE and all-cause death was increased in patients with prior IE (*p* < 0.001), but not associated with age (*p* = 0.50), sex (*p* = 0.67) or complexity of disease (*p* = 0.59). While there was no difference in events over the entire observational time period (*p* = 0.22), the time dynamics varied between TPVI and SPVR: Within the first year, the risk for IE and all-cause death was lower after TPVI (Hazard Ratio (HR) 95% CI 0.19 (0.06–0.63; *p* = 0.006) but increased over time and exceeded that of SPVR in the long term (HR 10.07 (95% CI 3.41–29.76; *p* < 0.001). **Conclusions**: Patients with TPVI appear to be at lower risk for early but higher risk for late IE, resulting in no significant difference in the overall event rate compared to SPVR. The results highlight the importance of long-term specialized care and preventive measures after both interventions.

## 1. Introduction

In patients with pulmonary valve dysfunction, valve replacement can be performed either surgically (SPVR) or through a transcatheter implantation (TPVI) approach. Since its introduction in 2000 [1], various analyses have shown an excellent long-term outcome for TPVI compared to SPVR [2,3,4]. Specifically in patients with congenital heart disease (CHD), a cohort of patients that faces repeated surgical interventions throughout their lifetime, TPVI appears to be an attractive minimally invasive alternative to repeated surgery. 

Beyond procedural considerations, patients with prosthetic valves are at risk for long-term complications, including infective endocarditis (IE) which is a feared adverse event as it may have a detrimental impact on patients’ morbidity and mortality [5,6]. In CHD patients, the incidence of IE has been reported to be higher compared to the general population [7], and case fatality rates continue to range between 3 and 17% [8,9,10].

While previous studies have addressed this issue in single center analyses as well as cohorts from multiple specialized centers, we aimed to assess the frequency of IE after pulmonary valve replacement based on a nationwide administrative dataset reflecting a non-selected group of patients. Furthermore, we aimed to identify predictors for its occurrence and to compare the outcome of TPVI versus SPVR focusing on IE and a composite endpoint of IE and all-cause death. 

## 2. Materials and Methods

Data were obtained retrospectively and covered the period between the 1st of January 2005 and the 31st of December 2018. The dataset was based on administrative information provided by the BARMER health insurance company that includes ≈9.2 million insurance holders. Previous data have shown that the administrative database of the BARMER health insurance is representative of the German population with statutory health insurance (data on file). All cardiac and extracardiac in- and out-of-hospital diagnoses are encoded in the dataset as documentation is obligatory for reimbursement in Germany. Diagnoses are documented using the German Modification of the International Statistical Classification of Diseases and Related Health Problems, 10th Revision (ICD-10-GM) and procedures with German Procedure Classification (OPS) codes. 

All CHD patients who received TPVI (i.e., Melody, Edward Sapien) or SPVR (allo-, xeno- or mechanic valve) were included in the analysis. The exact type of implanted TPVI or SPVR (manufacturer/material) cannot be identified due to a lack of granularity of the German OPS codes. Only patients with underlying CHD and in whom pulmonary valve replacement was performed without other relevant additional surgical interventions (that may potentially raise risk for IE) were included in the analysis. As such, only closure of an atrial or ventricular septal defect was allowed as concomitant surgeries. The analysis was restricted to patients older than four years of age to ensure a patient size where both treatment modalities would be technically feasible (see Figure S1, Supplementary Materials for a detailed flow-chart of the selection process). SPVR and TPVI implantations were marked as index events, and patients were screened for previous diagnoses of IE coded in the database. Patients’ baseline characteristics and comorbidities were assessed at time of the index event (for a full list of ICD-10-GM codes, OPS procedure codes and ATC drug codes, see Table S1, Supplementary Materials). The relevant ICD-10-GM codes for congenital heart disease are displayed in Table S2, Supplementary Materials. The patients were followed after the index event and censored if they underwent another pulmonary valve replacement or left the insurance company during follow-up. The pre-specified primary endpoints of the study were IE and a composite endpoint of IE and all-cause death. As for anonymity of insurance data, no prior written informed consent for the analyzed data had to be obtained. This study was approved by the local Ethics Committee (OptAHF project/2018-579-f-S) and was conducted in accordance with the Declaration of Helsinki.

### Statistical Analysis

Categorical variables are presented as absolute numbers and percentages, while continuous variables are shown as median and interquartile ranges (IQR). Differences between the groups were assessed by a Fisher’s exact test or a Mann–Whitney U test depending on data type.

For the time-to-event analysis, we fitted a multivariable Cox regression model that adjusted for patient characteristics at the time of the index event. As the effect on the endpoints changed over time, which goes against the basic assumption of proportional hazards, a step function on three time intervals ([index, 1 year), [1 year, 3 years), [3 years, ∞)) was used. Thus, the problem of non-proportional hazards was avoided [11,12]. Through this method, the changing effect of the parameters potentially influencing the outcome of the valve type over time was accounted for. The model further used a robust variance estimator to adjust for any similarities between data points from the same patient. 

All analyses were explorative, and a two-sided *p*-value < 0.05 was considered significant throughout the study. All analyses were performed using the statistical software R version 4.0.3. (R foundation, Vienna, Austria), the survival-package in particular.

## 3. Results

### 3.1. Patient Demographics and Pulmonary Valve Replacement

**Figure 1:**
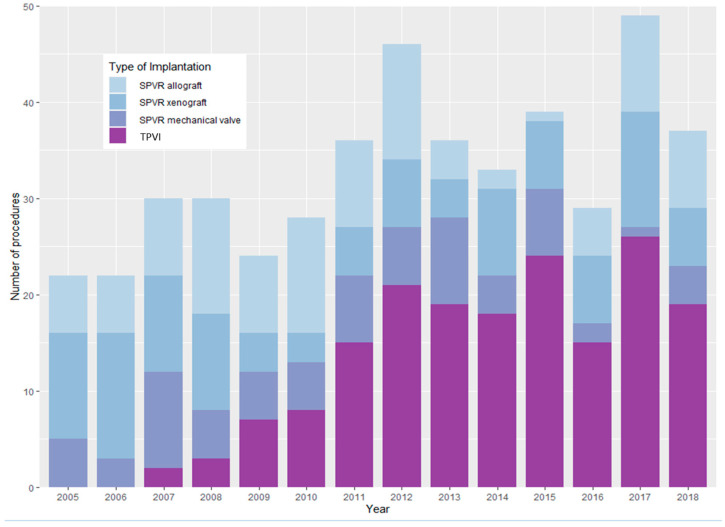
Number of pulmonary valve implantations during the study period stratified by calendar year and treatment modality (transcatheter pulmonary valve implantation = TPVI, surgical pulmonary valve replacement = SPVR).

Overall, 549 patients received an isolated pulmonary valve replacement within the study period from 2005 to 2018. Of those, 413 CHD patients (58.4% male, median age 18.9 (IQR 12.3–33.4)) underwent a total of 461 procedures and fulfilled the criteria for inclusion in the analysis. Of these, 34.4% (*n* = 142) were treated with TPVI, whilst 65.6% (*n* = 271) underwent SPVR for their first procedure. Of the patients treated with SPVR, 36.5% (*n* = 99) received an allograft implantation, 38.0% (*n* = 103) a xenograft and 25.5% (*n* = 69) a mechanical valve. As shown in Figure 1, there was an increase in TPVI throughout the time of analysis, whilst the usage of SPVR decreased.

Patient demographics stratified into patients undergoing TPVI versus SPVR are displayed in Table 1. 

Overall, there was a slight preponderance of male patients at the time of valve replacement that did not differ relevantly between the two cohorts (60.6% (*n* = 86) in TPVI versus 57.2% (*n* = 155) in SPVR; *p* = 0.53). Patients with TPVI were significantly older compared to patients undergoing SPVR (median 21.9 years (IQR 13.9–33.6) versus 17.0 years (IQR 10.6–32.9); *p* = 0.008). Arterial hypertension (*p* = 0.007) and obesity (*p* < 0.001) were observed more frequently in patients after TPVI. Table 1 additionally provides information about underlying CHD diagnoses, with the most prevalent being Tetralogy of Fallot (54.5%; *n* = 225).

### 3.2. Pulmonary Valve Re-Replacement

**Figure 2:**
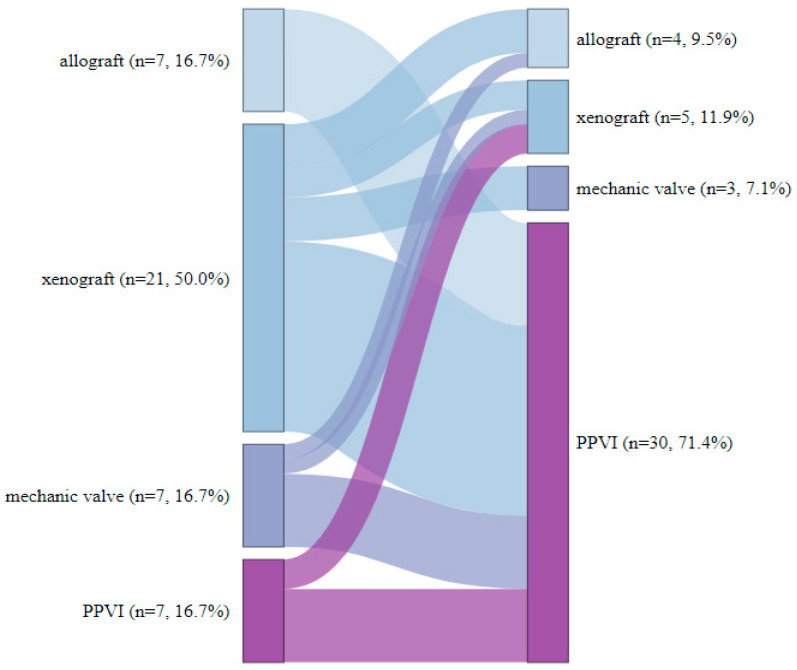
Distribution of surgical procedures for the initial and subsequent surgery (*n* = 42).

Over a median follow-up of 3.8 years (IQR 1.6–7.1), 10.2% (*n* = 42) of patients required pulmonary valve re-replacement (7 from the TPVI group and 35 from the SPVR group). Of these, six patients required multiple surgeries. For re-replacement, the preferred method was TPVI in 71.4% (*n* = 30), whilst 28.6% (*n* = 12) received SPVR. Figure 2 illustrates the distribution of patients categorized by the type of heart valve during the initial surgical intervention and upon subsequent re-operation.

Overall, the median time to second intervention was 4.6 years (IQR 2.2–7.3 years). There were no significant differences regarding time to second intervention between the TPVI and SPVR groups (median 4.6 years (IQR 1.1–5.6) TPVI versus median 5.2 years (IQR 2.6–7.8) SPVR; *p* = 0.26). 

### 3.3. Predictors and Rates of IE and of IE and All-Cause Death after Pulmonary Valve Implantation

Over a median follow-up period of 3.5 (1.4–6.8) years, IE occurred in 8.0% (*n* = 37) of cases. Irrespective of implantation method, a prior diagnosis of IE was associated with a higher risk of post interventional IE (Hazard Ratio (HR) 2.74; 95% CI 1.13–6.63; *p* = 0.025) and the composite of IE or all-cause death (HR 3.63; 95% CI 1.71–7.70; *p* < 0.001). Patient age (IE *p* = 0.17, composite endpoint *p* = 0.50), sex (IE *p* = 0.34, composite endpoint *p* = 0.67) or complexity of congenital heart disease (IE *p* = 0.71, composite endpoint *p* = 0.59) were not significantly associated with IE as well as IE and all-cause death after pulmonary valve replacement (see Table 2 for Cox regression analyses). 

**Figure 3:**
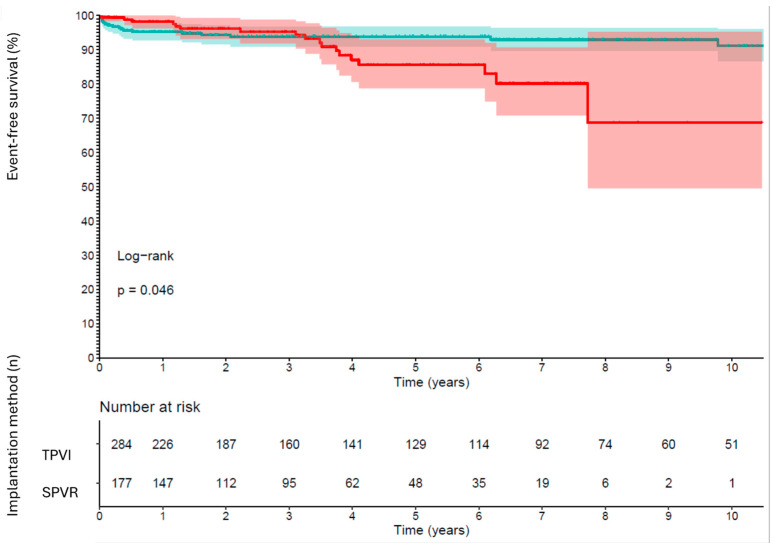

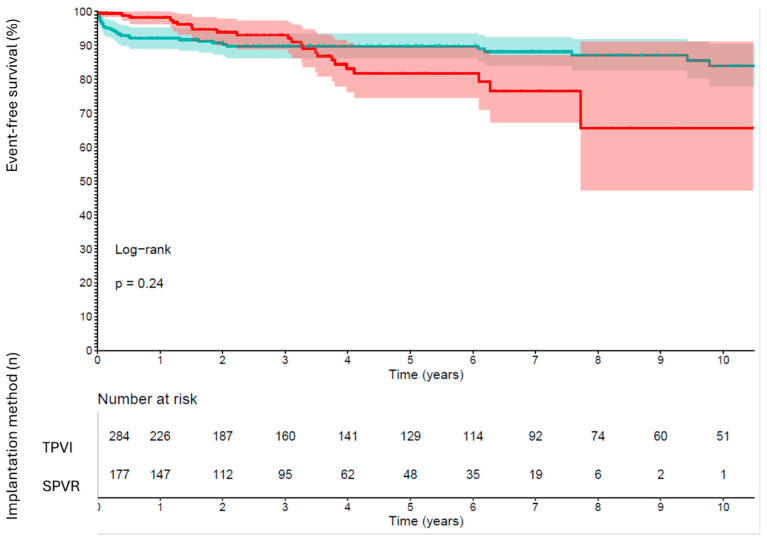
Kaplan–Meier estimates for infective endocarditis and combined endpoint of infective endocarditis and death (red = transcatheter pulmonary valve implantation (TPVI), green = surgically implanted pulmonary valves (SPVR)).

Over the entire observational time period, risk for IE (*p* = 0.22) as well as IE or all-cause death (*p* = 0.66) were similar for TPVI and SPVR. There were, however, marked differences in the timing of occurrences of the endpoints depending on the implantation method: within the first year after pulmonary valve intervention, risk for IE and all-cause death was significantly lower after TPVI compared to SPVR (HR 0.19; 95% CI 0.06–0.63; *p* = 0.006). On the long-term assessment, however, risk increased significantly for TPVI compared to SPVR (HR 10.07; 95% CI 3.41–29.76; *p* < 0.001) (see Table 2). These time-dynamics were consistent with the Kaplan–Meier survival estimates for both endpoints, where the percentages for event-free survival were higher in the TPVI cohort within the first years, but after approximately three years, the probabilities reversed in TPVI compared to SPVR (see Figure 3). Consequently, after approximately three years, patients with TPVI were at higher risk for IE than SPVR. After six years, only a few patients in the TPVI group were left for the estimates. 

The Kaplan–Meier estimates stratified by type of valve (i.e., allo-, xeno- and mechanic valve replacement) are displayed in the online Supplementary Materials (Figure S2a,b).

After a diagnosis of IE, 8% (*n* = 3) received re-replacements within 90 days and 5% (*n* = 2) of cases in whom IE was documented died within 30 days. All these patients had originally been treated with SPVR. Table 3 provides descriptive details on the cases diagnosed with IE stratified by initial treatment modality. 

As the only additional significant difference between both cohorts of patients with a diagnosis of IE, the timing of the diagnosis became overt (median 3.4 years (IQR 1.2–3.9) in TPVI versus 0.3 years (IQR 0.1–1.5); *p* = 0.007) in SPVR. 

## 4. Discussion

Our data give novel insights into the time dynamics of infective endocarditis after pulmonary valve replacement in congenital heart disease patients. Specifically, they show that patients treated with a transcatheter pulmonary valve implantation are at a lower risk for early endocarditis specifically in the first year compared to patients with surgically implanted valves. Over time, however, risk increases gradually and after three years, the probability of IE reverses with the result of an elevated long-term risk for infective endocarditis and all-cause death after transcatheter valve implantation in comparison to patients that have primarily been treated surgically. However, the overall net burden of endocarditis and all-cause death is comparable between patients receiving either treatment strategy. Our findings emphasize the need for long-term specialized follow-up and awareness on behalf of treating clinicians specifically after transcatheter valve implantation. This approach should be supplemented by ongoing patient education about meticulous dental hygiene and regular dental care as well as awareness of the early signs of IE in this vulnerable patient population.

In our nationwide analysis, the overall frequency of IE after pulmonary valve replacement was 8% at a median follow-up of 3.5 years without relevant differences between the surgical and interventional cohort. This is broadly comparable to previous reports with incidences ranging between 3 and 17% [2,10,13,14,15,16]. The discrepancies may be explained by the different median time of follow-up as well as differences in patient composition of the analyzed groups. Previous head-to-head comparisons between TPVI and SPVR regarding the risk of IE were largely single-center-based [2,4,13]. We contend that our large analysis adds to the literature by providing a non-selective, real-world nationwide overview of the overall risk of new IE comparing TPVI with SPVR. 

A previous diagnosis of IE was associated with de novo IE in our analysis. Other parameters such as complexity of congenital heart disease, age or gender did not emerge as risk factors. As for small number of events, robust predictive parameters are yet to be determined. An identification of risk factors would enable clinicians to identify high-risk subgroups that benefit from early identification and treatment. A relatively large multicenter analysis that includes patients with TPVI only support our results as they found an association with IE and prior IE with a hazard of 2.2 [15]. 

As a novel aspect adding to the current literature, our data reveal insights into the time of occurrence of IE after pulmonary valve replacement. We found that patients after TPVI were at a lower risk for early endocarditis whilst the long-term risk for prosthetic-valve endocarditis increased over time and even exceeded the risk of patients with SPVR. In the current literature, early endocarditis which occurs within the first year after implantation is distinguished from late endocarditis, which is defined as IE at least one year after implantation, because their microbiological profiles differ [17]. The rationale is that in early IE, most frequently, nosocomial infections are the cause, whilst late IE is mainly caused by non-nosocomial microorganisms. In early IE, most often, coagulase-negative Staphylococci are found, whilst in late IE, Streptococci viridans are frequently found [17]. Potentially, the higher number of early IEs in SPVR is caused by a longer in-hospital stay, longer treatment in an intensive care unit, and a higher number of drains and indwelling catheters that can cause bloodstream infections. A late IE, which occurs more frequently in the TPVI cohort, is more likely to be non-nosocomial. Amat-Santos et al. identified dental, respiratory and skin infections as the main pathogens for IE in patients with TPVI [18]. 

Apart from microbiological differences, there is an ongoing discussion on the relevance of tissue characteristics and implantation technique as predictors for IE [10,13,19,20].

For transcatheter valves, most frequently, Melody valves (Medtronic, Dublin, Ireland) are used [5,13]. These transcatheter valves are manufactured from bovine jugular veins. The other less frequently used valve type in the pulmonary position is the Edward Sapien valve (Edwards Lifesciences, Irvine, CA, USA), which is constructed from bovine pericardium tissue. A meta-analysis comparing Melody to Edwards Sapien valves reported a higher incidence of IE in the Melody cohort [21]. In a recent multicenter analysis on endocarditis after transcatheter valve implantation, however, the type of transcatheter valve was not a predictor for IE in a multivariable analysis [10]. 

As for bovine jugular vein grafts, Sharma et al. compared surgically implanted Contegra bioprosthetic-valved conduits, which are glutaraldehyde-preserved valve-containing bovine jugular vein grafts (Contegra, Medtronic Inc., Minneapolis MN, USA) to Melody valves [19]. No difference in the occurrence of IE between both patient cohorts was found. Stammitz et al. support these results, as they showed that for bovine jugular vein valves in general, the risk for IE is increased compared to other types of pulmonary valves irrespective of the method of deployment based on nationwide data from a German registry of CHD patients [22]. These results suggest that the valve material rather than the implantation technique relate to the susceptibility for microorganisms and risk of IE. As our data show differences in time dynamics, potentially, there is a difference in the susceptibility to certain nosocomial or non-nosocomial microorganisms depending on the type of valve. 

In vitro studies indicated that traumatic manipulation (‘crimping’) at the time of implantation of the transcatheter valves may cause micro damage to the valve surface, which may predispose for IE [20]. Although speculative, our data of higher long-term risk for IE after TPVI may be explained through this manipulation. These damages may confer a higher susceptibility of the valve surface for IE in TPVI over time, when degenerative changes occur and further affect the surface of the implanted valve. 

Given the overall balanced impact on early and late IE between the treatment modalities, the obvious clinical implications of our findings are less focused on the choice of treatment strategy. The decision to use a transcatheter or surgical valve implantation/replacement approach should be guided by anatomical aspects, surgical risk and the technical feasibility of TPVI. In addition, the most favorable option should be discussed in a team fashion between interventional cardiologists and cardiac surgeons [23]. 

Beyond the implantation period, our analysis emphasizes on the importance of long-term prevention measures in everyday clinical practice such as stringent dental hygiene, adequate antibiotic prophylaxis to avoid IE, as well as early detection and appropriate treatment of the condition. As such, our data suggest that no patient after TPVI/SPVR should be discharged from regular specialized cardiac follow-up and especially that patients need to constantly be reminded of the persistent and potentially increasing risk for IE over time after TPVI. 

### Limitations

This study is based on a large dataset of unselected administrative data. Due to the mandatory reimbursement system in Germany, the data are complete and cover all relevant codes. Coding errors and misclassifications may occur, but major events such as IE are unlikely to be missed or coded in error. The retrospective design and general constraints in the use of administrative care data have been described previously [24]. Due to the nature of the underlying data, clinical parameters such as hemodynamic values, laboratory and microbiological findings, or overall clinical morbidity were not available for analysis and could not be incorporated into the predictive models. Due to a lack of granularity of the German OPS codes, the exact type of pulmonary valve implanted is unknown and cannot be identified based on our data. Additionally, although information about the re-replacement of pulmonary valves has been gathered in our analysis, no conclusions about the durability of the implanted valves can be drawn as the indication for valve re-replacement remains unclear and the decision is ultimately at the treating physicians’ discretions. 

## 5. Conclusions

According to our non-selective, nationwide administrative data including 461 pulmonary valve implantations, transcatheter pulmonary valve implantation is the increasingly preferred method in clinical practice. Overall, no relevant differences between occurrences of endocarditis in patients with transcatheter versus surgically implanted valves was noted. However, there were marked differences in the time of occurrence of endocarditis: whilst the risk for early endocarditis was lower after TPVI compared to surgically implanted valves and there was a significant difference regarding the composite endpoint of endocarditis and death, the risk increased and exceeded that of SPVR in the long run. Our data emphasize the importance of awareness of the long-term risk for IE. Educational management of congenital heart disease patients about the importance of primary preventive measures is a life-long requirement and needed even many years after valve replacement.

## Figures and Tables

**Table 1:** Patient demographics overall and stratified by treatment modality (transcatheter pulmonary valve implantation = TPVI, surgical pulmonary valve replacement = SPVR).

Parameter	First Procederes *n* = 413	TPVI *n* = 142	SPVR *n* = 271	*p*-Value
Type of valve at index, *n* (%)			Allograft 99 (36.5) Xenograft 103 (38.0) Mechanic 69 (25.5)	
Age at time of surgery, median years (IQR)	18.9 (12.3–33.4)	21.9 (13.9–33.6)	17.0 (10.6–32.9)	0.008
Time to re-intervention, median years (IQR)	4.6 (2.2–7.3)	4.6 (1.1–5.6)	5.2 (2.6–7.8)	0.26
Sex, male, *n* (%)	241 (52.3)	86 (60.6)	155 (57.2)	0.53
Principal diagnosis, *n* (%)				0.008
Tetralogy of Fallot	225 (48.8)	66 (46.5)	159 (58.7)	
Transposition of the great arteries	32 (6.9)	14 (9.9)	18 (6.6)	
Univentricular circulation	52 (11.3)	20 (14.1)	32 (11.8)	
Valvular disease	27 (5.9)	17 (12.0)	10 (3.7)	
Others	77 (16.7)	28 (19.7)	52 (19.2)	
Down syndrome, *n* (%)	10 (2.2)	1 (0.7)	9 (3.3)	0.18
Immunodeficiency, *n* (%)	33 (7.2)	14 (9.9)	19 (7.0)	0.34
Cancer diagnosis, *n* (%)	19 (4.1)	8 (5.6)	11 (4.1)	0.47
Previous myocardial infarction, *n* (%)	4 (0.9)	2 (1.4)	2 (0.7)	0.61
History of heart failure, *n* (%)	181 (39.3)	70 (49.3)	111 (41.0)	0.12
History of cardiac arrhythmias, *n* (%)	173 (37.5)	68 (47.9)	105 (38.7)	0.08
Previous stroke, *n* (%)	5 (1.1)	0 (0.0)	5 (1.8)	0.17
Arterial hypertension, *n* (%)	75 (16.3)	36 (25.4)	39 (14.4)	0.007
Obesity, *n* (%)	44 (9.5)	29 (20.4)	15 (5.5)	<0.001
Smoking history, *n* (%)	19 (4.1)	7 (4.9)	12 (4.4)	0.81
Psychological and behavioral disorders, *n* (%)	283 (61.4)	102 (71.8)	181 (66.8)	0.32
History of alcohol abuse, *n* (%)	11 (2.4)	3 (2.1)	8 (3.0)	0.76
Diabetes, *n* (%)	19 (4.1)	10 (7.0)	9 (3.3)	0.14
Chronic kidney disease (severe), *n* (%)	1 (0.2)	1 (0.7)	0 (0.0)	0.34
Hepatic fibrosis/cirrhosis, *n* (%)	1 (0.2)	0 (0.0)	1 (0.4)	1.00

**Table 2:** Multivariable Cox regression analyses for (a) infective endocarditis (IE) and combined endpoint (b) infective endocarditis or all-cause death (transcatheter pulmonary valve implantation = TPVI, surgical pulmonary valve replacement = SPVR).

(a)		
IE	Hazard Ratio (95% Confidence Interval)	*p*-Value
Prior Endocarditis	2.74 (1.13–6.63)	0.025
Younger than 18 years of age	1.62 (0.81–3.24)	0.17
Sex, female	0.70 (0.34–1.45)	0.34
More than simple congenital heart disease	0.78 (0.21–2.94)	0.71
TPVI versus SPVR (time-period of assessment)		
0–1 year	0.38 (0.11–1.27)	0.12
1–3 years	2.21 (0.48–20.21)	0.31
3–10 years	21.80 (4.65–101.95)	<0.001
(b)		
IE and all-cause death	Hazard Ratio (95% Confidence Interval)	*p*-Value
Prior Endocarditis	3.63 (1.71–7.70)	<0.001
Younger than 18 years of age	0.82 (0.47–1.44)	0.50
Sex, female	1.13 (0.65–1.95)	0.67
More than simple congenital heart disease	0.76 (0.29–2.04)	0.59
TPVI versus SPVR (time-period of assessment)		
0–1 year	0.19 (0.06–0.63)	0.006
1–3 years	2.08 (0.64–6.82)	0.225
3–10 years	10.07 (3.41–29.76)	<0.001

**Table 3:** Overview of the cases of congenital heart disease patients with infective endocarditis overall and stratified by treatment modality (transcatheter pulmonary valve implantation = TPVI, surgical pulmonary valve replacement = SPVR).

Parameter	Overall Patients	TPVI	SPVR	*p*-Value
Infective endocarditis	37	18 (10.2)	19 (6.7)	0.22
Infective endocarditis and all-cause death	56	23 (13.0)	33 (11.6)	0.66
Parameter at time of IE				
Age at time of surgery, median years (IQR)	15.2 (10.7–23.2)	14.0 (11.5–20.2)	15.3 (10.3–25.0)	0.61
Age at time of IE, median years (IQR)	16.9 (IQR 14.9–24.5)	17.0 (15.8–24.0)	15.5 (10.6–25.8)	0.26
Time between surgery and IE, median years (IQR)	1.3 (0.2–3.7)	3.4 (1.2–3.9)	0.3 (0.1–1.5)	0.007
Length of in-hospital stay, median days (IQR)	31 (9–46)	35 (16–51)	29 (8–45)	0.69
Death				
-within 30 days	2 (5.0)	0 (0%)	2 (11%)	0.49
-within 90 days	2 (5.0)	0 (0%)	2 (11%)	0.49
Valve replacement				
-within 30 days	0 (0)	0 (0%)	0 (0%)	1.00
-within 90 days	3 (8.0)	0 (0%)	3 (16%)	0.23

## Data Availability

The insurance data are protected by the German data protection laws (‘Bundesdatenschutzge-setz’, BDSG). As for data protection laws, the uncensored data cannot be made available in the manuscript, the supplemental files or a public repository. They are stored on a secure drive at the Barmer Health Insurance Research Institute to facilitate replication of the results. Generally, access to data of statutory health insurance companies for research purposes is possible only under the conditions defined in German Social Law (SGB V § 287). Requests for data access can be sent as a formal proposal specifying the recipient and purpose of data transfer to the appropriate data protection agency. Access to the data used in this study can only be provided to external parties under the conditions of the cooperation contract of this research project and after written approval by the health insurance fund.

## Supplementary Materials

The following supporting information can be downloaded at https://www.mdpi.com/article/10.3390/jcm13092683/s1, Figure S1: Flow-chart of the selection process and cohort definition. Table S1: ICD-10-GM codes/OPS diagnosis and procedure codes relevant for analyses. Table S2: ICD-10-GM Codes used for identification and grouping of patients with congenital heart disease (CHD). Figure S2: Kaplan–Meier estimates stratified by type of pulmonary valve for (a) infectious endocarditis and (b) combined endpoint of infectious endocarditis and death (TPVI = transcatheter pulmonary valve implantation).
