# Supplementary material for: Occurrence and Outcome of Infective Endocarditis after Surgical Compared to Transcatheter Pulmonary Valve Implantation in Congenital Heart Disease
Source: J Clin Med. 2024 May 2;13(9):2683. doi: 10.3390/jcm13092683 (PMC11084703; doi:10.3390/jcm13092683)

Supplementary online only material

Figure S1: Flow-chart of the selection process and cohort definition.

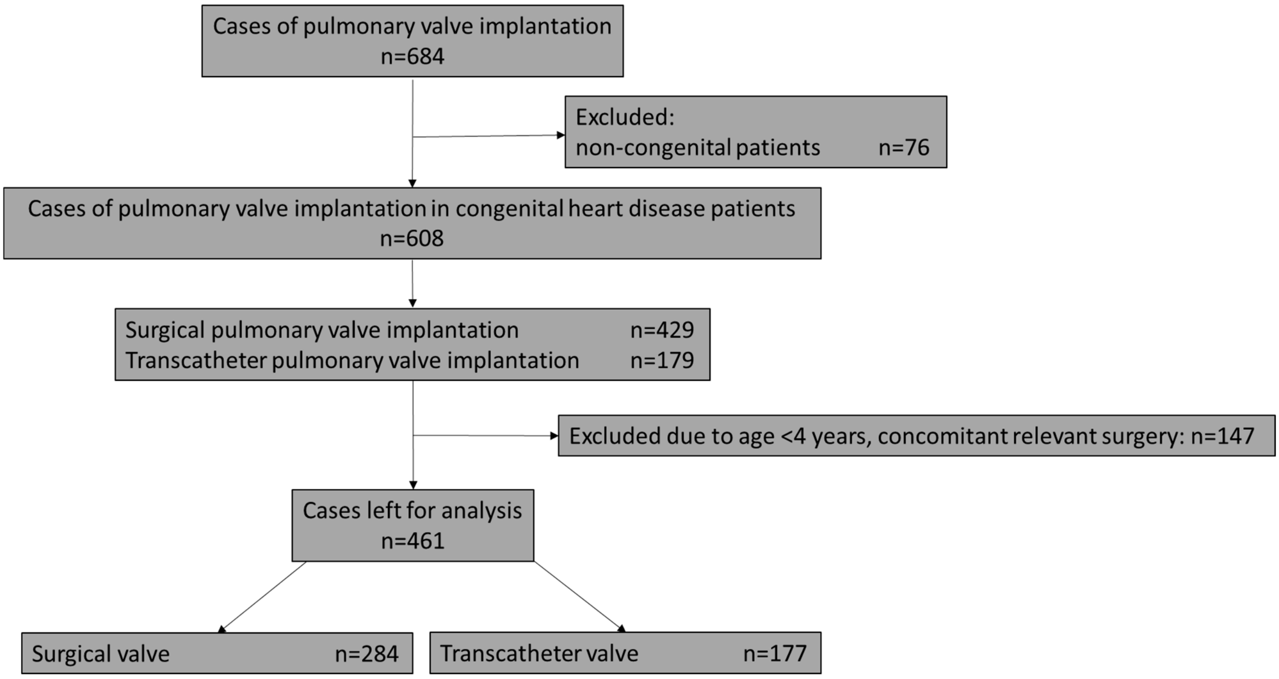

Table S1: ICD-10-GM Codes/ OPS diagnosis and procedure codes relevant for analyses.

Parameter	Codes
Type of pulmonary valve implantation	OPS:
Pulomonary allograft implantation	5-358.21, 5-351.31, 5-358.29, 5-351.37
Pulmonary xenograft implantation	5-358.22, 5-351.32, 5-351.33
Pulmonary mechanic valve implantation	5-358.24, 5-358.25, 5-351.34
Percutaneus pulmonary valve implantation	5-35a.1
Concomitant surgeries	OPS:
subvalvular fibrous/muscular resection	5-354.22, 5-354.23
supravalvular resection	5-354.24
Total correction of a Tetralogy of Fallot, double outlet right ventricle, correction of absent pulmonary valve syndrome	OPS: 5-359.0, 5-359.30, 5-359.8
Aortic valve surgery	OPS: 5-358.0, 5-350.0, 5-350.1, 5-351.0, 5-353.0, 5-353.6, 5-353.7, 5-354.0
Mitral valve surgery	OPS: 5-358.1, 5-350.2,5-350.3, 5-351.1, 5-353.2, 5-354.1
Coronary artery bypass graft	OPS: 5-36
Atrial/ventricular septal defect correction	OPS: 5-356
Down syndrome	ICD: Q90
Immunodeficiency	ICD: D80-D90
Cancer diagnosis	ICD: C00-C97
Myocardial infarction	ICD: I21, I 22
Heart failure	ICD: I50, I43, I42
Cardiac arrhythmias	ICD: I47-I49
Stroke	ICD: I63, I64
Arterial hypertension	ICD: I10, I11, I12, I13, I15
Obesity	ICD: E66
Smoking	ICD: F17
Psychatric and behavioral disorders	ICD: F00-F98, F10, K70, T51.0, T51.9, K29.2, K86.0, I42.6, K85.2
Alcohol abuse	ICD: G62.1, G72.1, E24.4
Diabetes	ICD: E10-E14
Chronic kidney disease (severe)	ICD: N18.4, N18.5
Hepatic fibrosis/cirrhosis	ICD: K72.1, K72.7
Endocarditis	ICD: I33, I38, I39.3

Table S2: ICD-10 GM-Codes used for identification and grouping of patients with congenital heart disease (CHD).

**Simple CHD**	
Isolated ventricular septal defect	Q21.0
Persistent arterial duct	Q25.0
Isolated congenital valve disease	Q23.0, Q23.1, Q22.4, Q22.8, Q22.9, Q23.2, Q23.3, Q22.1, Q22.2, Q22.3
Other congenital malformation of the great arteries	Q25.8, Q25.9
**Moderate complexity CHD**	
Tetralogy of Fallot	Q21.3, Q21.80, (Q22.0 and Q21.0)
Ebstein’s anomaly	Q22.5
Aortic isthmus stenosis, interrupted aortic arch	Q25.1, Q25.2
Atrioventricular septal defect	Q21.2
Partial anomalous pulmonary venous connection	Q26.3, Q26.4
**Severely complex CHD**	
Univentricular heart	Q20.1, Q20.2, Q20.4, Q22.6, Q23.4, (Q22.0 without Q21.0)
Eisenmenger’s syndrome	I27.8 and at least one further Q-Code with the exception of Q21.1<br><br>or Q21.88 and at least one further Q-Code with the exception of Q21.1
Transposition of the great arteries (TGA)	Q20.3, Q20.5
Other complex heart malformation, e.g. total anomalous pulmonary venous connection, common arterial trunk	Q20.0, Q26.2

Figure S2 Kaplan-Meier estimates stratified by type of pulmonary valve for a) infectious endocarditis and the b) combined endpoint of infectious endocarditis and death (TPVI= transcatheter pulmonary valve implantation).

a)

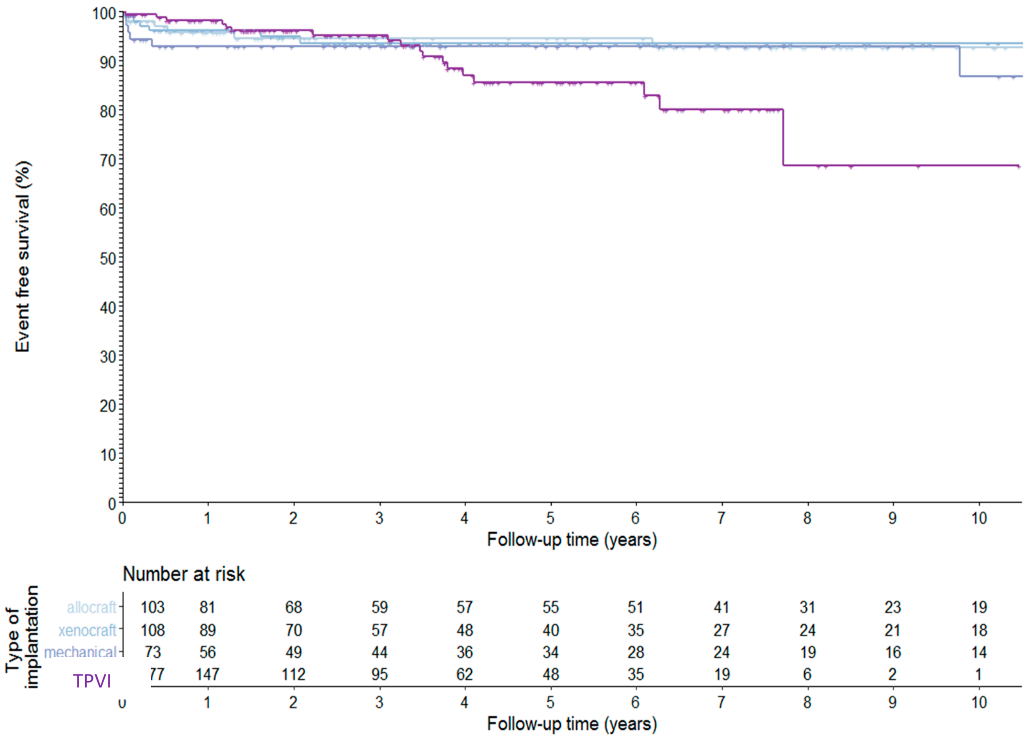

b)

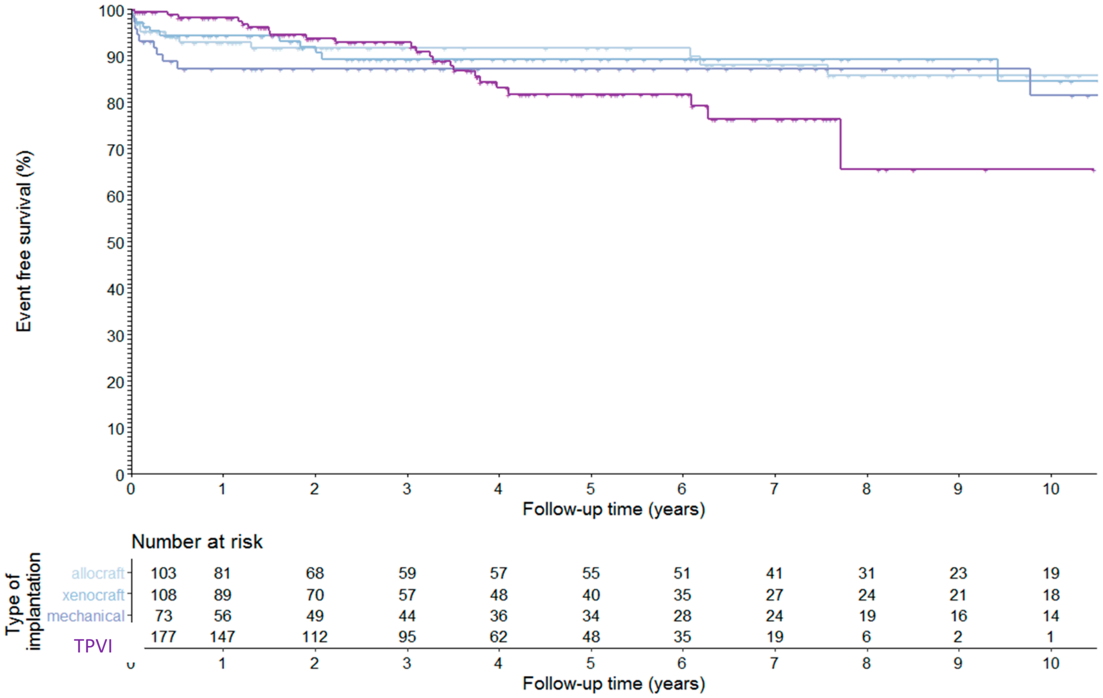

Supplement: Supplementary file 1 [file jcm-13-02683-s001.zip › jcm-2966087-supplementary.pdf]
